# The forestecology R package for fitting and assessing neighborhood models of the effect of interspecific competition on the growth of trees

**DOI:** 10.1002/ece3.8129

**Published:** 2021-09-29

**Authors:** Albert Y. Kim, David N. Allen, Simon P. Couch

**Affiliations:** ^1^ Program in Statistical & Data Sciences Smith College Northampton MA USA; ^2^ Biology Department Middlebury College Middlebury VT USA; ^3^ Department of Biostatistics Johns Hopkins University Baltimore MD USA

**Keywords:** forest ecology, ForestGEO, interspecific competition, neighborhood competition, R, spatial cross‐validation, tree growth

## Abstract

1. Neighborhood competition models are powerful tools to measure the effect of interspecific competition. Statistical methods to ease the application of these models are currently lacking.
2. We present the forestecology package providing methods to (a) specify neighborhood competition models, (b) evaluate the effect of competitor species identity using permutation tests, and (cs) measure model performance using spatial cross‐validation. Following Allen and Kim *(PLoS One, 15,* 2020, e0229930), we implement a Bayesian linear regression neighborhood competition model.
3. We demonstrate the package's functionality using data from the Smithsonian Conservation Biology Institute's large forest dynamics plot, part of the ForestGEO global network of research sites. Given ForestGEO’s data collection protocols and data formatting standards, the package was designed with cross‐site compatibility in mind. We highlight the importance of spatial cross‐validation when interpreting model results.
4. The package features (a) tidyverse‐like structure whereby verb‐named functions can be modularly “piped” in sequence, (b) functions with standardized inputs/outputs of simple features sf package class, and (c) an S3 object‐oriented implementation of the Bayesian linear regression model. These three facts allow for clear articulation of all the steps in the sequence of analysis and easy wrangling and visualization of the geospatial data. Furthermore, while the package only has Bayesian linear regression implemented, the package was designed with extensibility to other methods in mind.

## Graphical abstract

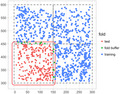

## 1 INTRODUCTION

Repeat‐censused forest plots offer excellent opportunities to test neighborhood models of the effect of competition on the growth of trees (Canham et al., 2004). Neighborhood models of competition have been used to test whether the species identity of a competitor matters Uriarte et al. (2004); measure species‐specific competition coefficients (Das, 2012; Tatsumi et al., 2016); test competing models to see what structures competitive interactions, for example, traits or phylogeny (Allen & Kim, 2020; Uriarte et al., 2010); and inform selective logging practices (Canham et al., 2006). Although these are well‐described methods, few methods are currently available for easy application.

We address this shortcoming with the forestecology R package providing methods and data for forest ecology model fitting and assessment, available on CRAN (https://cran.r‐project.org/package=forestecology) and on GitHub (https://github.com/rudeboybert/forestecology). The package is written to model stem diameter growth between two censuses based on neighborhood competition, largely following the methods in Allen and Kim (2020).

Let *i* = 1,…, *n_j_* index all *n_j_* trees of “focal” species *j*; let *j* = 1,…, *J* index all *J* focal species; and let *k* = 1,…, *K* index all *K* “competitor” species. The average annual growth in diameter at breast height (DBH) *y_ij_* (in centimeters/year) of the *i* ^th^ tree of focal species *j* is modeled as

(1) $$\begin{array}{c} y_{\mathrm{ij}}=\beta_{0,j}+\beta_{\text{dbh},j}\cdot {\text{dbh}}_{\mathrm{ij}}+\sum_{k=1}^{K}\lambda_{\mathrm{jk}}\cdot x_{\mathrm{ijk}}^{\text{comp}}+\varepsilon_{\mathrm{ij}} \end{array}$$

where *β*_0,_ *_j_* is the diameter‐independent growth rate of species *j*; *dbh_ij_* is the DBH of the focal tree at the earlier census and *β*_dbh,_ *_j_* the slope of that species's diameter–growth relationship; $x_{\mathrm{ijk}}^{\text{comp}}$ is the sum of some numerical explanatory variable of all trees of competitor species *k*, and *λ_jk_* quantifies the corresponding change in growth for individuals of species *j* from these competitors; and *ε_ij_* is a random error term distributed Normal (0, *σ* ^2^).

Allen and Kim (2020) use the sum of the basal area of all trees of competitor species *k* as $x_{\mathrm{ijk}}^{\text{comp}}$. Furthermore, they estimate all parameters via Bayesian linear regression, while exploiting Normal/Inverse Gamma conjugacy to derive closed‐form solutions to all posterior distributions.^1^ These closed‐form solutions are not as computationally expensive as approximations from Markov chain Monte Carlo algorithms.

To evaluate whether competitor species identity matters, Allen and Kim (2020) run a permutation test where a null hypothesis of no species grouping‐specific effects of competition is assumed; thus, the species identity of all competitors can be permuted:

(2) $$\begin{array}{c} \;H_{0}:\lambda_{\mathrm{jk}}=\lambda_{j}\;\text{for}\;\text{all}\;k=1,\ldots ,K\\ \text{vs}.\;H_{A}:\text{at}\;\text{least}\;\text{one}\;\lambda_{\mathrm{jk}}\;\text{is}\;\text{different} \end{array}$$

Furthermore, to account for the spatial autocorrelation in their estimates of out‐of‐sample model error, Allen and Kim (2020) use spatial cross‐validation. Estimates of model error that do not account for this dependence tend to underestimate the true model error (Roberts et al., 2017).

The package is designed with “tidy” design principles in mind (Wickham et al., 2019). Much like all tidyverse packages, forestecology has verb‐named functions that can be modularly composed using the pipe %>% operator to sequentially complete all necessary analysis steps (Bache & Wickham, 2020).

Furthermore, the inputs and outputs of most functions use the same “simple features for R” data structures for spatial data from the sf package (Pebesma, 2018). Previously, sp package classes were commonly used for storing spatial data and interfacing with geospatial libraries (Bivand et al., 2013); the sf package aims to improve on the sp package by:

1. Using simple feature access as the base standard for representing and encoding spatial data, rather than shapefiles (Herring, 2011).
2. Leveraging improvements in external libraries for reading and writing spatial data (GDAL) and for geometrical operations (GEOS) (GEOS Development Team, 2017; Warmerdam, 2008).
3. Integrating closely with the popular tidyverse suite of packages for data science (Wickham et al., 2019).

By using the sf package classes to represent spatial data rather than the sp package, the implementation and use of the forestecology package's spatial algorithms was greatly simplified.

## 2 forestecology WORKFLOW: A CASE STUDY

We present a case study of forestecology's functionality on data from the Smithsonian Conservation Biology Institute (SCBI) large forest dynamics plot in Front Royal, VA, USA, part of the ForestGEO global network of research sites (Anderson‐Teixeira et al., 2015; Bourg et al., 2013; Davies et al., 2021). The 25.6‐ha (640 × 400 m) plot is located at the intersection of three of the major physiographic provinces of the eastern United States—the Blue Ridge, Ridge and Valley, and Piedmont provinces—and is adjacent to the northern end of Shenandoah National Park.

The package has the following goals: to evaluate (a) the effect of competitor species identity using permutation tests and (b) model performance using spatial cross‐validation. We outline the four‐step basic analysis sequence:

1. Compute the growth of stems based on two censuses.
2. Add spatial information:
  - Define a buffer region of trees.
  - Add spatial cross‐validation block information.
3. Identify all focal trees and their competitors.
4. Apply model, which includes:
  - Fit model.
  - Compute predicted values.
  - Visualize posterior distributions.

We start by loading all packages (all code in this paper can be copied from here https://github.com/rudeboybert/forestecology/blob/master/paper/paper.R).

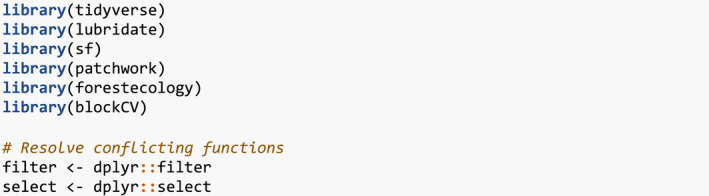

### 2.1 Step 1: Compute the growth of trees based on census data

We first compute the growth of trees using data from two censuses. compute_growth() computes the average annual growth based on census data that roughly follows ForestGEO standards. Despite such standards, minor variations will still exist between sites, thereby necessitating some data wrangling. For example, the SCBI site records all DBH values in millimeters (Bourg et al., 2013), whereas the Michigan Big Woods site used in Allen and Kim (2020) records them in centimeters (Allen et al., 2020).

We load both 2008 and 2014 SCBI census.csv files as they existed on GitHub on 2021/08/02 and perform minor data wrangling (Gonzalez‐Akre, McGregor, et al., 2020). We then only consider a 9‐ha subsection of the 25.6 ha of the site to speed up computation for this example: gx from 0–300 instead of 0–400 and gy from 300–600 instead of 0–640.

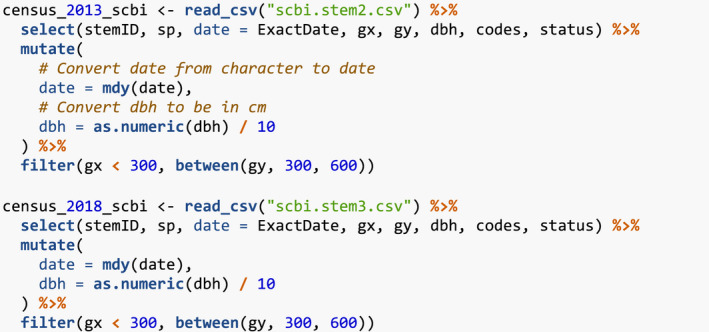

These two data frames are then used as inputs to compute_growth(), along with id specifying the variable that uniquely identifies each tree‐stem. We also discard all resprouts with code == R in the later census, since we are only interested in the growth of surviving, and not resprouted, stems.

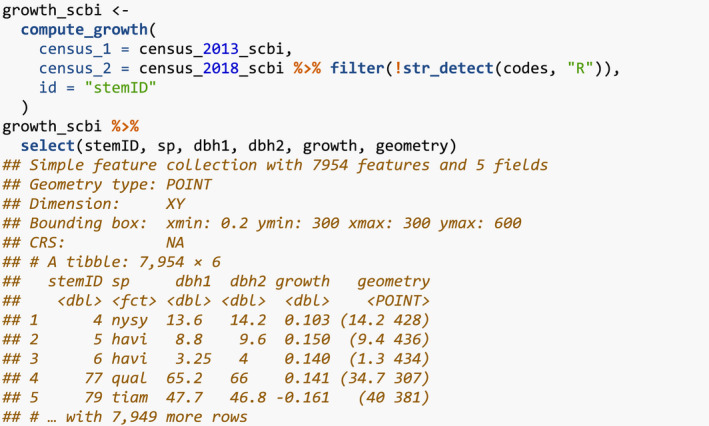

**FIGURE 1:**
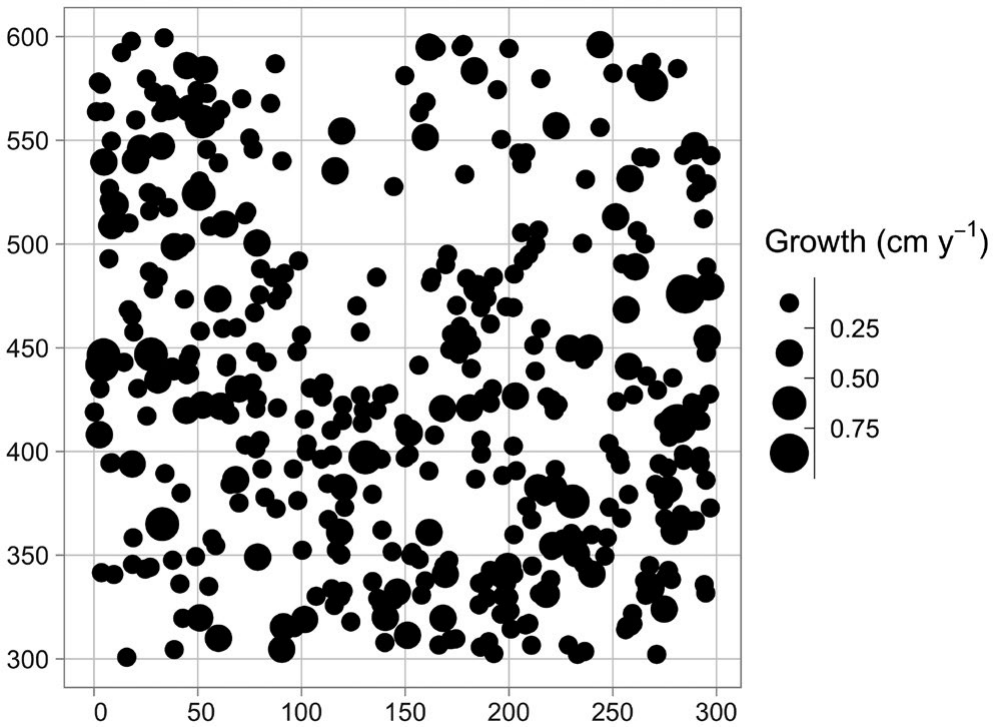
Step 1—Compute growth of trees based on census data. A map of the growth of a random sample of 500 trees from a 9‐ha subsection of the Smithsonian Conservation Biology Institute (SCBI) forest plot

The output growth_scbi is a data frame of class sf that includes among other variables the species variable sp converted to a factor, the average annual growth in DBH (cm·y^−1^) for all stems that were alive at both time points, and the sf package's encoding of geolocations of geometry type <POINT>. Given that growth_scbi is of class sf, it can be easily plotted in ggplot2 using geom_sf() as seen in Figure 1.

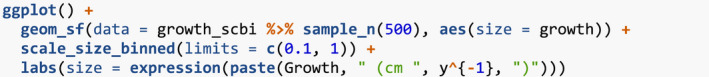

We also load species information as it existed on GitHub on 2021/08/02, which includes family, genus, and species information, as well as classifications of the canopy position (canopy, canopy emergent, understory, shrub layer), drought tolerance (intolerant, resistant), and other characteristics of the species.

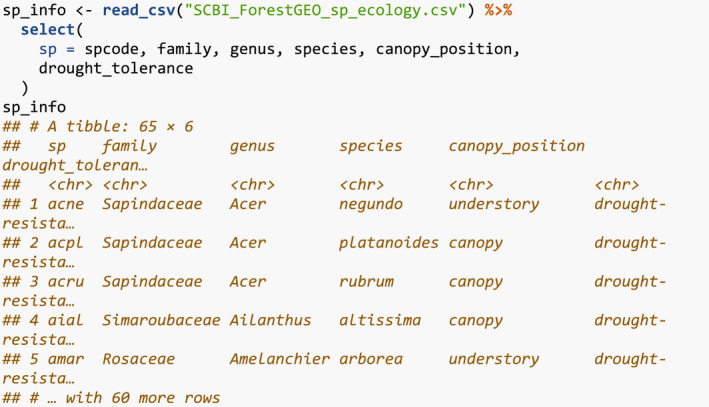

We join this species information to our growth_scbi data frame and convert the species variable to a factor.

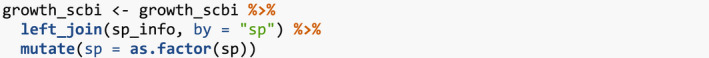

Furthermore, we compute two potential competitor explanatory variables $x_{\mathrm{ijk}}^{\text{comp}}$ from Equation 1. First, the basal area of each tree as a function of its DBH in the earlier census. Second, the aboveground biomass as estimated by allometric equations encoded in the get_biomass() function from the allodb package (Gonzalez‐Akre et al., 2020); this function has DBH, species, and geographic coordinates as arguments.

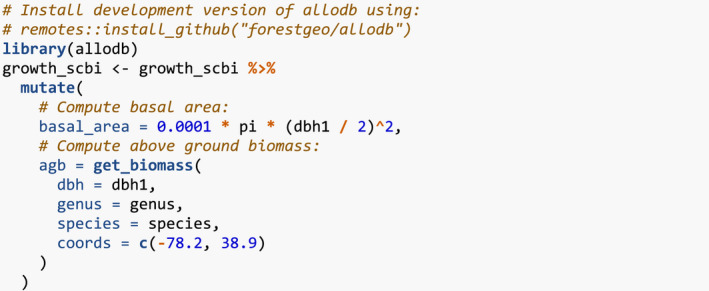

### 2.2 Step 2: Add spatial information

We then add spatial information to growth_scbi. We first add a “buffer region” to the periphery of the study region. Since some of our model's explanatory variables are cumulative, we must ensure that all trees being modeled are not biased to have different neighbor structures. This is of concern for trees at the boundary of the study region who will not have all their neighbors included in the census stems. To account for such edge effects, only trees that are not part of this buffer region, that is, are part of the interior of the study region, will have their growth modeled (Waller & Gotway, 2004).

Our model of interspecific competition relies on a spatial definition of who competitor trees are: all trees within a distance comp_dist of a focal tree. Here, we set comp_dist to 7.5m, a value informed by other studies (Canham et al., 2004; Canham et al., 2006; Uriarte et al., 2004), but the package could also be used to compare multiple distances and see which is best supported (see Appendix 1). We use comp_dist and a manually constructed sf representation of the study region's boundary as inputs to add_buffer_variable() to add a buffer Boolean variable to growth_scbi. All trees with buffer equal to FALSE will be our focal trees whose growth will be modeled, whereas those with TRUE will only act as competitor trees.

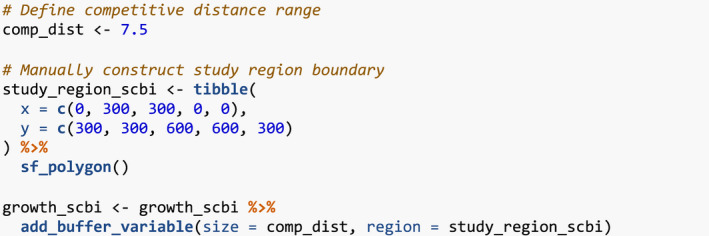

The second element of spatial information we add are blocks corresponding to folds of a spatial cross‐validation algorithm. Conventional cross‐validation algorithms assign individual observations to folds by randomly resampling them all while assuming they are statistically independent. In the case of forest census data however, observations exhibit spatial autocorrelation. We therefore incorporate this dependence into the cross‐validation algorithm by resampling spatial blocks of trees (Pohjankukka et al., 2017; Roberts et al., 2017).

We first manually define an sf object defining four folds that partition the study region. We then use the output of the spatialBlock() function from the blockCV package to associate each tree in growth_scbi to the correct foldID (Valavi et al., 2019). This foldID variable will be used in Section 2.6.

**FIGURE 2:**
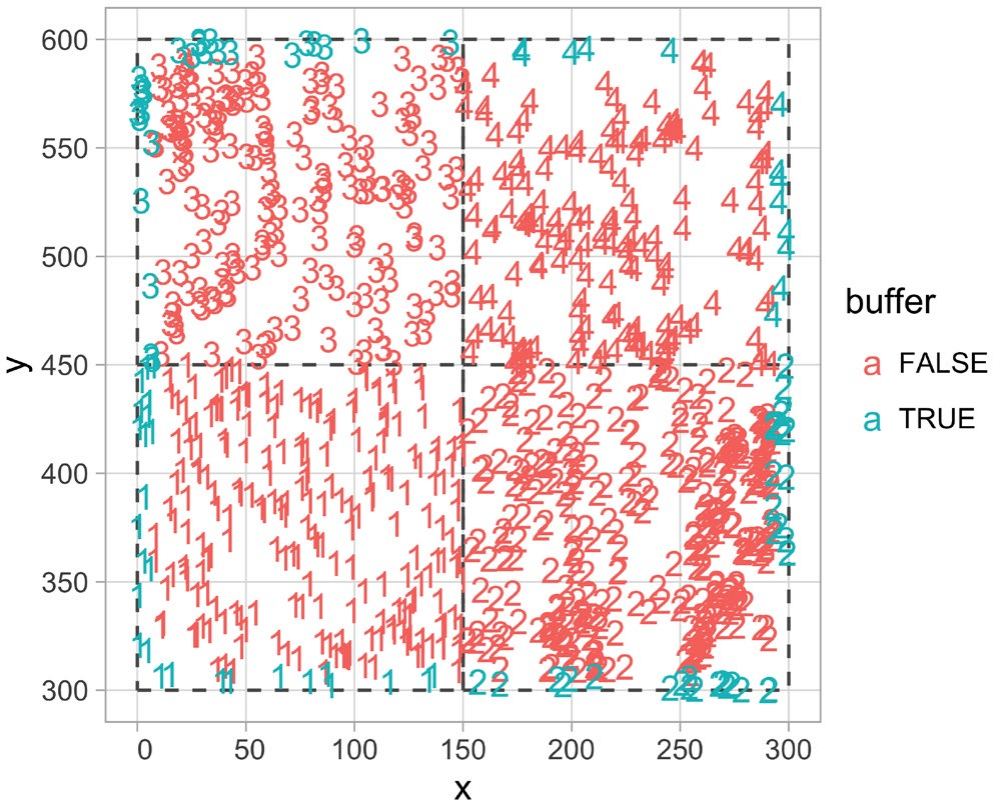
Step 2—Add spatial information. A buffer region and spatial cross‐validation blocks 1 through 4. The location of each tree is marked with its fold number where the folds are delineated with solid lines. The color of each digit indicates whether the tree is part of the buffer region (thus will only be considered as a competitor tree) or is part of the interior of the study region (thus is a focal tree whose growth is of modeled interest)

Figure 2 illustrates the net effect of adding these two elements of spatial information to growth_scbi.

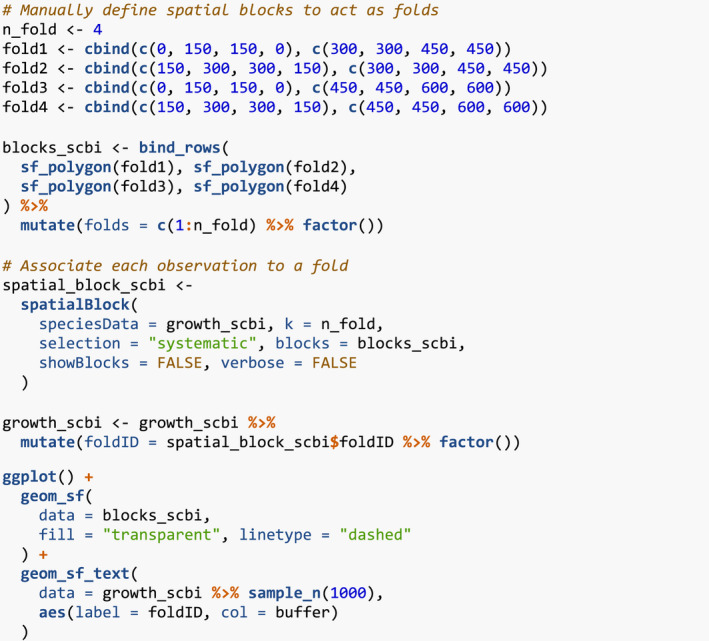

### 2.3 Step 3: Identify all focal and corresponding competitor trees

We then identify all focal trees and their corresponding competitor trees and, more specifically, identify all trees that are not part of the buffer region, have a valid growth measurement, and have at least one neighbor within 7.5 m. We do this using create_focal_vs_comp(), which takes the previously detailed comp_dist and id arguments, the sf representation of the spatial cross‐validation blocks blocks_scbi, and a specification comp_x_var of the basal_area variable we use as the competitor explanatory variable $x_{\mathrm{ijk}}^{\text{comp}}$ from Equation 1. This function returns a new data frame focal_vs_comp_scbi.

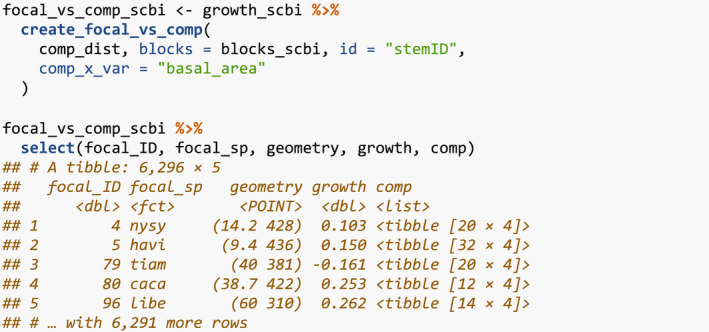

The resulting focal_vs_comp_scbi has 6,296 rows, representing the subset of the 7,954 trees in growth_scbi that will be considered as focal trees. The variables focal_ID and focal_sp relate to tree‐stem identification and species information. Most notably however is the variable comp, which contains information on all competitor trees saved in tidyr package list‐column format (Wickham, 2020). To inspect this information, we flatten the comp list‐column for the tree with focal_ID 4 in the first row, here a tibble [20 × 4], into regular columns using unnest() from the tidyr package.

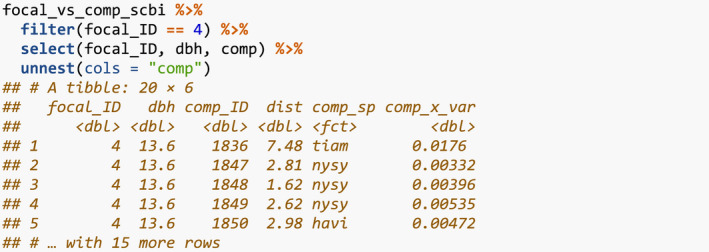

**FIGURE 3:**
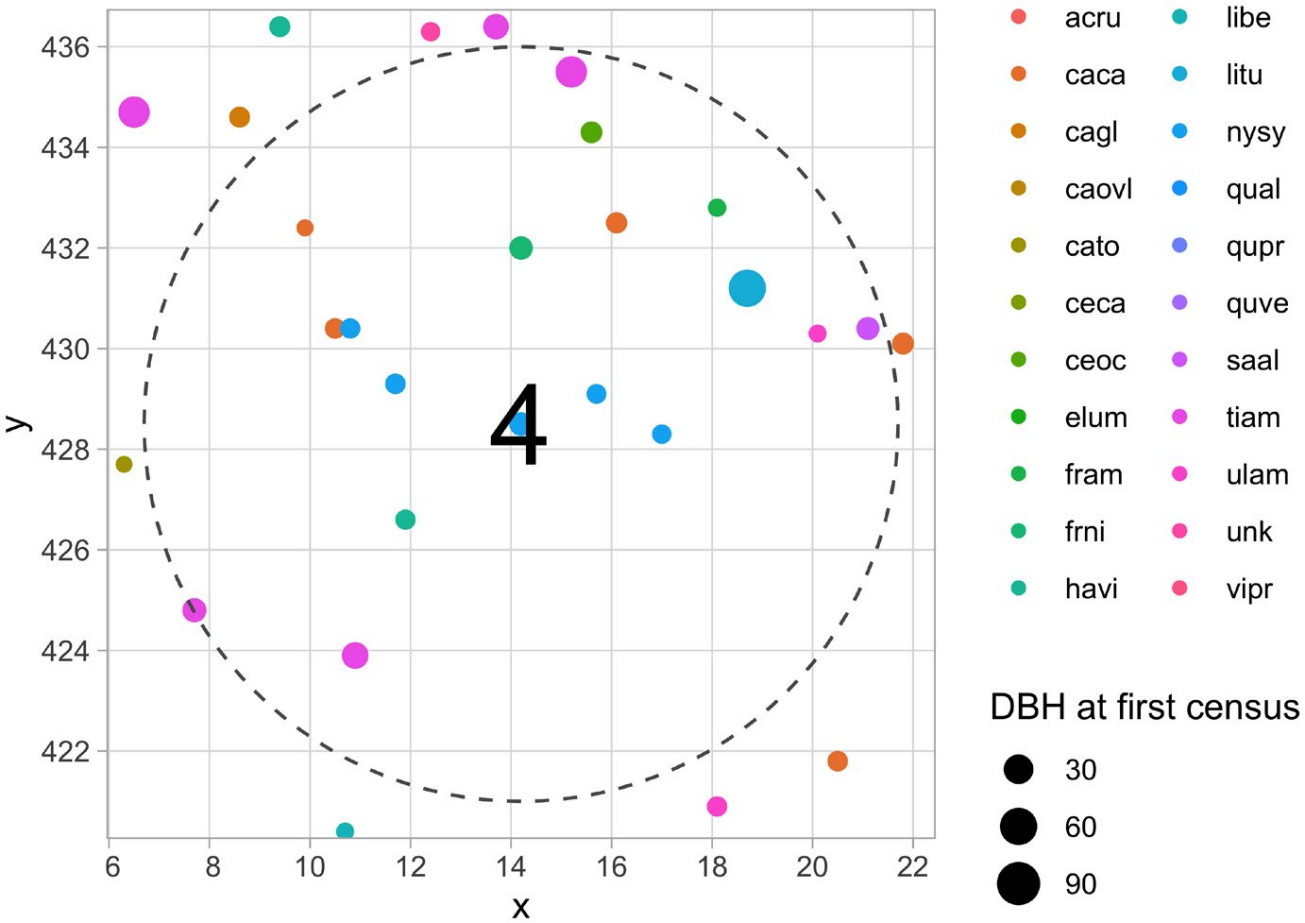
Step 3—Identify all focal and corresponding competitor trees. The dashed circle extends 7.5m away from the focal tree 4, while all 20 competitor trees are within this circle

We observe 4 variables describing 20 competitor trees: the unique tree‐stem ID, the distance to the focal tree (all ≤7.5 m), the species, and the basal area (in m^2^) calculated as $\frac{\pi \times {\left(\text{DBH}/2\right)}^{2}}{10000}$ for the DBH in cm from the earlier census. Saving competitor information in list‐column format minimizes redundancy since we do not need to repeat information on the focal tree 20 times. We visualize the spatial distribution of these trees in Figure 3.

Here, we use basal area as the continuous competitor explanatory variable, but the package is flexible to allow the user to specify any competitor explanatory variable (basal area, biomass, tree height, a soil nutrient value). The package can also be used to compare competitor explanatory variables and see which best explains tree growth, see Appendix 2 for an example comparing basal area and above‐ground biomass. Similarly, the package can use any categorical variable as an explanatory variable and compare between different categorical variables. For example, in Allen and Kim (2020), we compare grouping individuals based on species, family, and based on trait‐based groups. In Appendix 3, we give another example and compare grouping individuals by species or by potential canopy position (canopy, understory, shrub layer).

### 2.4 Step 4: Fit model

Lastly, we fit the competition Bayesian linear regression model for tree growth outlined in Equation 1 using comp_bayes_lm(). This function has an option to specify prior distributions of all parameters, chosen here to be the defaults detailed in ?comp_bayes_lm.

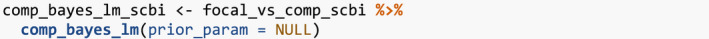

The resulting comp_bayes_lm_scbi is an object of S3 class type comp_bayes_lm containing the posterior values of all parameters. Furthermore, this class includes generics for three methods. First, the generic for print() displays the names of all prior and posterior parameters and the model formula:

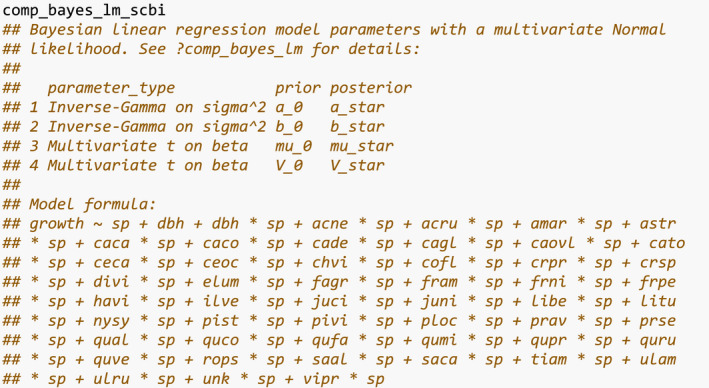

Next, the generic for predict() takes the posterior parameter values in comp_bayes_lm_scbi and a newdata data frame and outputs a vector growth_hat of predicted DBH values $\hat{y_{\mathrm{ij}}}$ computed from the posterior predictive distribution.

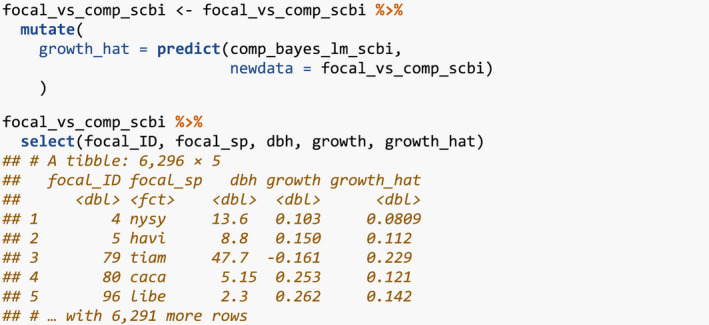

We can now compare the observed and predicted growths to compute the root mean squared error (RMSE) of our model:

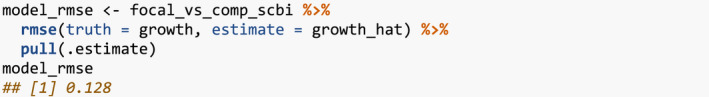

**FIGURE 4:**
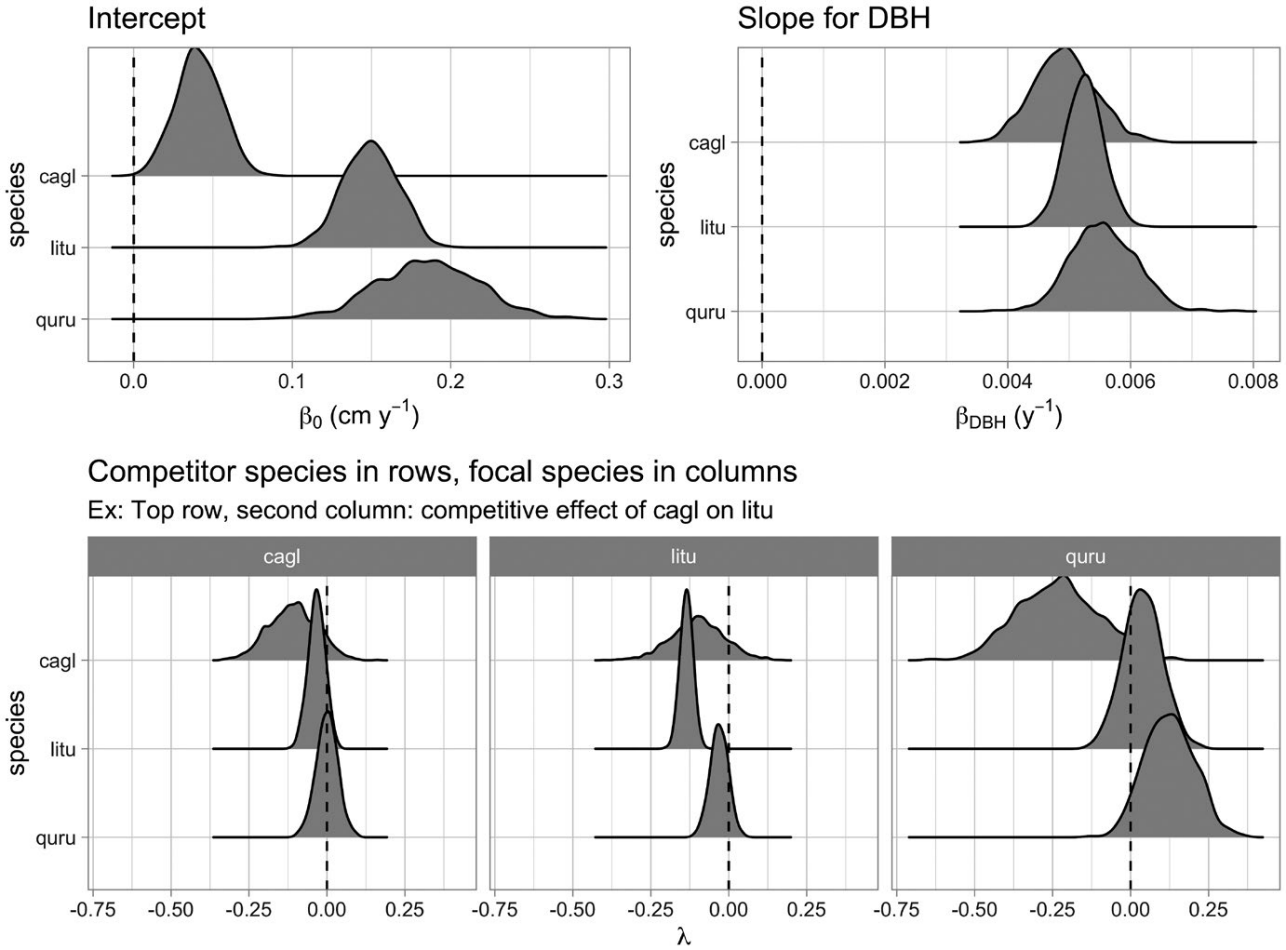
Step 4—Fit model. Posterior distributions of all parameters. For compactness, we include only three species

Lastly, the generic for ggplot2::autoplot() allows us to visualize all posterior distributions, as seen in Figure 4. Setting type to “intercepts” and “dbh_slopes” returns species‐specific posterior distributions for *β*_0,_ *_j_* and *β*_dbh,_ *_j_*, respectively, while setting type = “competition” returns competition coefficients *λ_j,k_*.

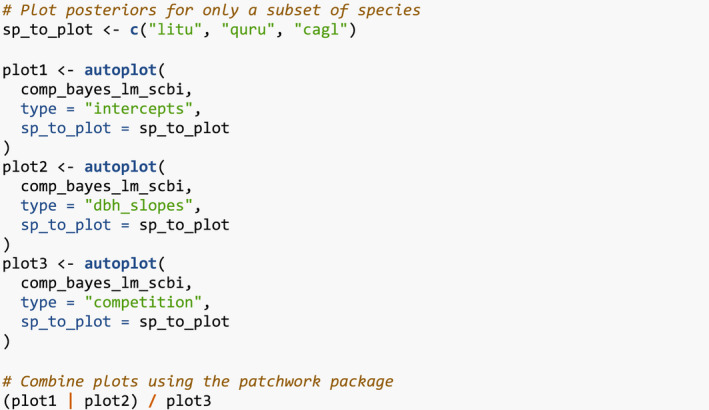

For many users, the visualizations of *λ_j,k_* will be of particular interest as they provide insight into species‐specific competitive interactions, where negative values indicate a competitor species which slows the growth of a focal species. Here, for example, we see that tulip poplars (litu) have a strong negative effect on the growth of conspecifics but relatively lesser effect on pignut hickory (cagl) and red oak (quru) neighbors.

Currently, the forestecology package can only fit the competition Bayesian linear regression model in Equation 1. However, it can be extended to any model as long as it is implemented in a function similar to comp_bayes_lm().

### 2.5 Evaluate the effect of competitor species identity using permutation tests

To evaluate the effect of competitor species identity, we use the above four steps along with the permutation test in Equation 2. Under a null hypothesis where competitor species identity does not matter, we can permute the competitor species identities within each focal tree, compute the RMSE test statistic, repeat this process several times to construct a null distribution, and compare it to the observed RMSE to assess significance. Going back to our example in Section 2.3 of focal tree with focal_ID 4 and its 20 competitors, the permutation test only randomly resamples the comp_sp variable without replacement, leaving all other variables intact. This resampling is nested within each focal tree in order to preserve neighborhood structure. We perform this permutation test once again using comp_bayes_lm() but by setting run_shuffle = TRUE.

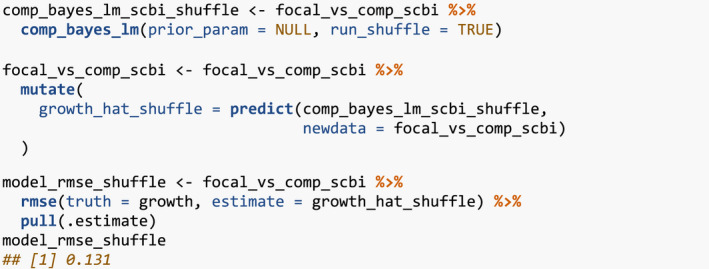

The resulting permutation test RMSE of 0.131 is larger than the earlier RMSE of 0.128, suggesting that models that do incorporate competitor species identity better fit the data.

### 2.6 Evaluate model performance using spatial cross‐validation

To evaluate model performance, we use spatial cross‐validation. The model fit in Section 2.4 uses the same data to both fit and assess model performance. Given the spatial‐autocorrelation of our data, this can potentially lead to overfit models (Roberts et al., 2017). To mitigate this risk, we use the spatial cross‐validation blocking scheme encoded in the foldID variable from Section 2.2 and visualized in Figure 2.

**FIGURE 5:**
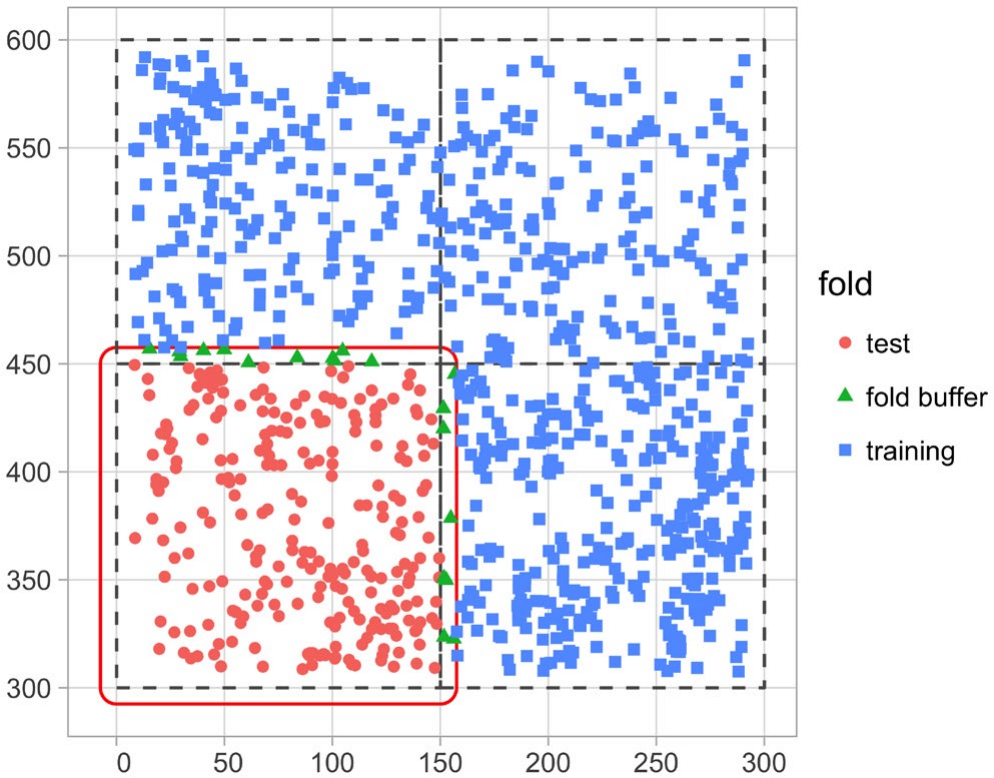
Schematic of spatial cross‐validation. Using the k = 1 fold (bottom‐left) as the test set, k = 2 through 4 as the training set, along with a fold buffer extending outward from the test set to maintain spatial independence between it and the training set

At each iteration of the cross‐validation, one fold acts as the test set and the remaining three act as the training set. We fit the model to all focal trees in the training set, apply the model to all focal trees in the test set, compute predicted values, and compute the RMSE. Furthermore, to maintain spatial independence between the test and training sets, a “fold buffer” that extends 7.5 m outward from the boundary of the test set is considered; all trees within this “fold buffer” are excluded from the training set (see Figure 5).

This process is repeated for each of the four folds acting as the test set, and then, the four RMSE’s are averaged to provide a single estimate of model error. This algorithm is implemented in run_cv(), which acts as a wrapper function to both comp_bayes_lm() that fits the model and predict() that returns predicted values.

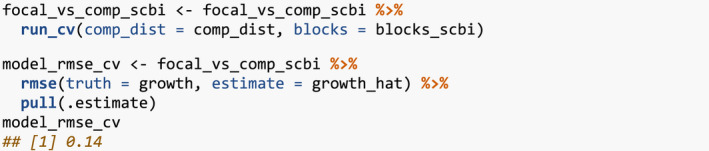

The resulting RMSE of 0.14 computed using cross‐validation is larger than the earlier RMSE of 0.128, suggesting that models that do not account for spatial autocorrelation generate model error estimates that are overly optimistic, that is, RMSE values that are too low.

## 3 IMPORTANCE OF SPATIAL CROSS‐VALIDATION

**FIGURE 6:**
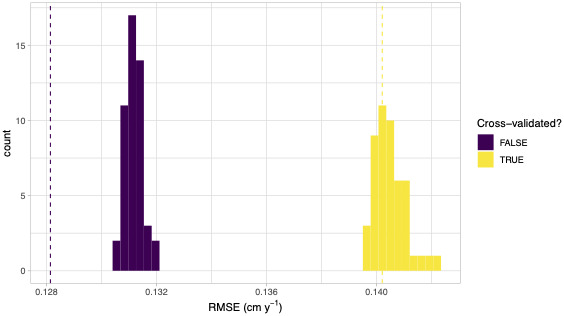
Comparison of root mean squared error of models for standard, permuted, and spatially cross‐validated error estimates. The dotted lines show observed RMSE while the histograms show the null distribution of RMSE for 49 permutations under the null hypothesis of no competitor species identity effects. The colors indicate whether spatial cross‐validation was used or not

run_cv() also accepts the run_shuffle argument in order to permute competitor species identity as described in Section 2.5. Figure 6 compares model performance for 49 permutations of competitor species and RMSE calculations, both with and without cross‐validation. Without cross‐validation, competitor species identity does matter as the observed RMSE was significantly lower than the permutation null distribution of RMSE. However, once we incorporate spatial cross‐validation, this improvement disappears. These results suggest that in this 9 ha subplot of the SCBI plot, competitive interactions do not depend on the identity of the competitor, which is the opposite of what has been observed in other locations (Allen & Kim, 2020; Uriarte et al., 2004). This provides a striking example of the importance of cross‐validation, as without it the over‐fit model gives rise to an incorrect conclusion.

**FIGURE 7:**
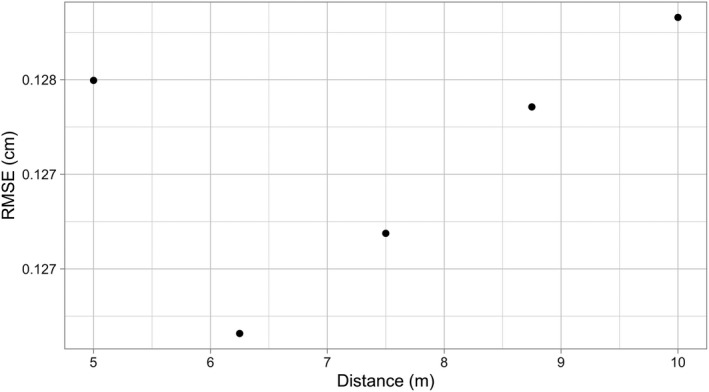
Cross‐validated RMSE estimates for 5 competitive distances

**FIGURE 8:**
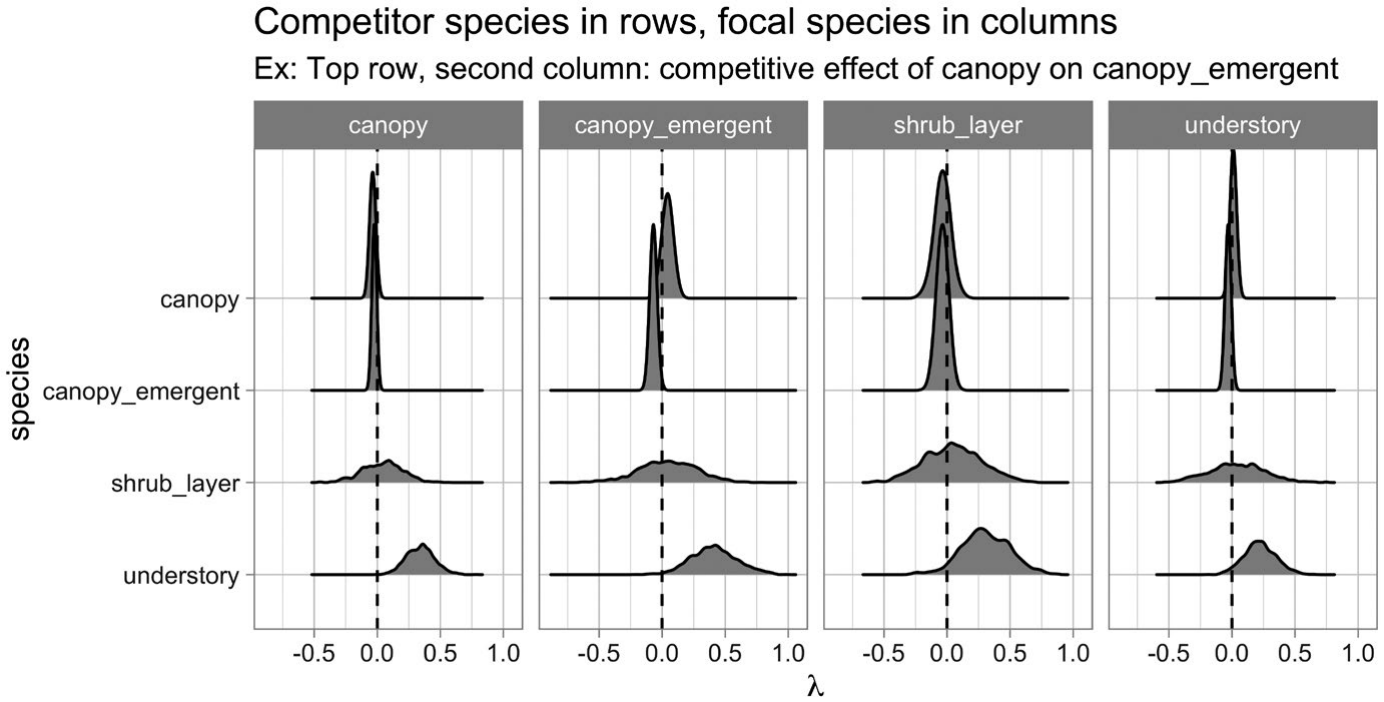
Posterior distributions of all competition parameters

## 4 CONCLUSION AND FUTURE WORK

The forestecology package provides an accessible way to fit and test models of neighborhood competition. The package follows the tidy data design principles, leverages the sf package for spatial data, and S3 open‐oriented model implementation structure (Pebesma, 2018). We hope that the package will increase the use of neighborhood competition models to better understand what structures plant competition.

While the package is designed with ForestGEO plot data in mind, we envision that it can be modified to work on any single large, mapped forest plot in which at least two measurements of each individual have been taken. Furthermore, we hope that future versions of the package will be flexible to other plot layouts, for example, inventory plot‐structure with many spatially separated plots like the US Forest Service Forest Inventory and Analysis plots (Smith, 2002).

We also hope to extend the forestecology package's functionality to account for a larger variety of models for tree growth. One clear future direction would be to allow competition based on species trait values rather than species identity. There is evidence that traits predict competitive outcomes (Kunstler et al., 2012; Lasky et al., 2014; Uriarte et al., 2010). Thus, an extension of the model would allow *λ* values from Equation 1 to be a function of the traits of competing species.

Lastly, the forestecology package current uses the blockCV package behind the scenes to create the spatial blocks acting as folds for our spatial cross‐validation algorithm detailed in Sections 2.2 and 2.6 (Valavi et al., 2019). This back‐end functionality could be substituted with the spatialsample package for spatial resampling infrastructure; a tidymodels package under active development as of 2021 (Kuhn & Wickham, 2020; Silge, 2021).

Lastly, currently, the package only implements the Bayesian linear regression model detailed in Equation 1. As we demonstrate in Section 2.4 however, the fitting of this model is self‐contained in a single function comp_bayes_lm() which returns an object of S3 class type comp_bayes_lm. This class has generic methods implemented to print, make predictions, and plot all results. Therefore, the package can be modularly extended to fit other models as long as they are coded similarly to comp_bayes_lm() and have equivalent generic methods implemented.

## CONFLICT OF INTEREST

We declare no competing interests.

## AUTHOR CONTRIBUTIONS

**Albert Y. Kim:** Conceptualization (equal); Methodology (equal); Software (equal); Writing‐original draft (lead); Writing‐review & editing (equal). **David N. Allen:** Conceptualization (equal); Methodology (equal); Software (equal); Writing‐original draft (supporting); Writing‐review & editing (equal). **Simon P. Couch:** Software (supporting); Writing‐review & editing (supporting).

### OPEN RESEARCH BADGES

This article has been awarded <Open Materials, Open Data> Badges. All materials and data are publicly accessible via the Open Science Framework at [https://zenodo.org/record/4070038; https://github.com/rudeboybert/forestecology/tree/master/paper].

## Data Availability

All data and source code for this manuscript on GitHub at https://github.com/rudeboybert/forestecology and are archived on Zenodo at https://doi.org/10.5281/zenodo.5367351. The example Smithsonian Conservation Biology Institute census and species information data are available on GitHub at https://github.com/SCBI‐ForestGEO/SCBI‐ForestGEO‐Data/tree/master/ and are archived on Zenodo at https://doi.org/10.5281/zenodo.2649301 (Gonzalez‐Akre, McGregor, et al., 2020).

## ENDNOTE

^1^
See Appendix 1, 2, 3, 4 of Allen and Kim (2020), available at https://doi.org/10.1371/journal.pone.0229930.s004.

## APPENDIX 1 Compare different competitive distances

For all the above analyses, we set the cutoff distance (comp_dist) for two stems to compete as 7.5m. This distance has been estimated in previous neighborhood competition studies in forests (Canham et al., 2004; Canham et al., 2006; Uriarte et al., 2004). We used 7.5 m in Allen and Kim (2020) as an average of the values estimated in other studies, but our package can be used to find which distance is best supported by the data. Here, we provide an example using another section of the SCBI plot to provide an additional example of the cross‐validation block layout. To speed computation, we do not consider species differences in competitive effects and treat all species as the same.

We observe in Figure 7, that a cutoff distance of approximately 6m minimizes the cross‐validation estimated RMSE.

## APPENDIX 2 Compare competitor explanatory variables

In the above code, we use the basal area of an individual as a continuous competitor explanatory variable, but the package allows the user to specify any competitor explanatory variable in the comp_x_var argument of create_focal_vs_comp function. Here, we use the cross‐validated model comparison to see which of two possible competitor explanatory variables computed in Section 2.1, basal area or aboveground biomass, best explains growth.

Here, we observe that basal area is a better competitor explanatory variable competitor explanatory variables $x_{\mathrm{ijk}}^{\text{comp}}$ from Equation 1 than aboveground biomass as suggested by the lower estimated RMSE.

## APPENDIX 3 Compare grouping variables

The package also allows the user to specify the categorical explanatory grouping variable. Here, we compare two different such variables: species and the potential canopy position of that species. If we had individual‐level crown classes (Smith (1986) dominant, codominant, intermediate and suppressed) that could also be used.

We find that species identity has a lower RMSE, so does a better job. We still however plot the competition posteriors for the canopy position groupings in Figure 8. Unsurprisingly, we see that canopy and canopy emergent competitors generally have negative effects on their neighbors, while shrubs and understory competitors have neutral or even positive effects.

## APPENDIX 4 Replicate RMSE comparison

This code replicates Figure 6: A comparison of root mean squared error of models for standard, permuted, and spatial cross‐validated error estimates.
